# The Influence of the Machining Drill and Direction of Rotation on the Surfaces of Ti6Al4V Dental Implants Subjected to Implantoplasty

**DOI:** 10.3390/jfb16060224

**Published:** 2025-06-16

**Authors:** Esteban Padullés-Gaspar, Francisco Real-Voltas, Esteban Padullés-Roig, Miguel Punset, Guillermo Cabanes, Pablo Fernández, Javier Gil

**Affiliations:** 1 Faculty of Dentistry, Universidad Internacional de Catalunya, Josep Trueta s/n, Sant Cugat del Vallés, 08195 Barcelona, Spain; cucoepg@uic.es (E.P.-G.); freal@uic.es (F.R.-V.); 2 Bioinspired Oral Biomaterials and Interfaces, Department of Materials Science and Engineering, Universitat Politècnica de Catalunya (UPC), Av. Eduard Maristany 16, 08019 Barcelona, Spain; a6r0e5s6@gmail.com; 3 Biomaterials, Biomechanics and Tissue Engineering, Department of Materials Science and Engineering, Universitat Politècnica de Catalunya (UPC), Av. Eduard Maristany 16, 08019 Barcelona, Spain; miquel.punset@upc.edu (M.P.); pablo.fernandez.gutierrez@upc.edu (P.F.); 4 Barcelona Research Centre in Multiscale Science and Engineering, Av. Eduard Maristany 10-14, 08019 Barcelona, Spain; 5 Department of Implantology, University of La Salle, EDE, c/Gaminedes 11, 28023 Madrid, Spain; guillermo@doctorcabanes.com

**Keywords:** peri-implantitis, implantoplasty, Ti6Al4V, titanium, dental implant, corrosion resistance, ion release

## Abstract

Implantoplasty is widely used to treat peri-implantitis by removing biofilms from Ti6Al4V dental implants using rotating drills. This study examined the effects of diamond and tungsten carbide drills, and rotation direction (clockwise/counterclockwise), on surface modification, corrosion behavior, and cytotoxicity. Machining was performed for one minute under a controlled load. Surface roughness, nanohardness, compressive residual stress, and wettability were evaluated, along with SEM and EDX microanalyses of the residues. Corrosion behavior was evaluated using potentiostatic and potentiodynamic tests in Hank’s solution. Ion release was monitored over time, and fibroblast viability was tested using extracts at various dilutions. The higher abrasiveness of diamond drills leads to increases roughness from 0.22 mm (control) to 0.73 and 0.59 for diamond and tungsten carbide drills, respectively; in hardness from 2.2 GPa for the control to 4.8 and 3.9 GPa; and in residual compressive stress from −26 to −125 and −111 MPa, with diamond drills inducing more significant changes and producing more hydrophilic surfaces with contact angles around 54° in relation to 80° and 62° for the control and tungsten carbide, respectively. Tungsten carbide drills caused lower corrosion rates (0.0323 mm/year) than diamond drills (0.052 mm/year). In addition, we observed the presence of tungsten ion release. Cytotoxic effects on human fibroblasts were observed with both bur types, and were more pronounced with tungsten carbide, especially at lower dilutions. Only 1:10 dilutions maintained consistent cytocompatibility. The rotation direction showed no significant impact. These findings emphasize the critical influence of bur selection in implantoplasty on the biological response of surrounding tissues.

## 1. Introduction

Over the past decade, peri-implantitis has emerged as one of the fastest-growing concerns in the field of oral implantology. This is caused by an inflammatory effect generated by bacterial colonization, in which proliferation and biofilm formation causes the loss of bone tissue [1,2,3]. The loss of bone generates a lack of mechanical fixation of the dental implant and can lead to fracture. Peri-implantitis is becoming a public health problem due to its high incidence in the population with dental implants. The European Societies of Periodontics values indicate that between 20 and 24% of dental implants placed should be revised before 10 years of placement [4,5,6,7].

In cases where infection is present and significant bone loss has occurred, the recommended approach typically involves removal of the infected dental implant, thorough decontamination of the surrounding tissues, and subsequent placement of a new implant [8,9,10]. However, in certain cases, bone regeneration is required, for which calcium phosphate-based materials are employed to stimulate new bone formation. Once sufficient bone tissue has regenerated, a new dental implant can be placed. Nevertheless, this regeneration process is time-consuming, and the clinician may opt to place a wider-diameter dental implant, utilizing the site of the extracted implant and achieving primary stability by anchoring it to the cortical bone. However, this approach often compromises adjacent teeth, sometimes requiring the extraction of healthy teeth to accommodate the new implant [11,12].

An alternative approach frequently employed in dental clinics is implantoplasty. This procedure involves mechanically removing biofilms from the infected surface of dental implants using rotary instruments to ensure complete decontamination. However, this process reduces the implant’s diameter, compromising its mechanical integrity as a consequence of the reduction in its load-bearing cross-section, including both static strength and fatigue resistance. Additionally, the metallic debris generated during machining is dispersed into the oral cavity, and not all particles are effectively aspirated. Residual particles remaining in the physiological environment have been shown in various studies to exhibit considerable cytotoxic effects, particularly in Ti6Al4V alloy implants [13,14,15,16].

Kotsakis et al. [17] demonstrated that implantoplasty procedures induce an inflammatory response, leading to a significant reduction in oxygen levels within the surrounding environment. This hypoxic condition selectively favors the elimination of aerobic bacteria while promoting the survival of anaerobic species, which are considered the most pathogenic for bone tissue. Oxygen depletion leads to a reduction in the titanium oxide (TiO_2_) layer, which serves as a protective passive film on dental implants, converting it into metallic titanium, while the released oxygen is absorbed by the surrounding tissues [17]. Once the inflammation subsides, oxygen can re-enter, allowing the titanium to oxidize. However, it does not form stoichiometric titanium dioxide; instead, non-stoichiometric mixed oxides are produced, which have been shown to exhibit incomplete cytocompatibility [18,19].

Although the drawbacks of implantoplasty are well recognized, its simplicity and practicality, particularly in elderly patients, have led to its routine application in clinical practice. To date, the influence of this procedure on the corrosion performance of machined dental implant surfaces, as well as on the dynamics of metal ion release into the physiological environment and adjacent peri-implant tissues, remains poorly understood. Moreover, the potential influence of the type of rotary instruments used and the direction of rotation on the implant surface characteristics has not yet been thoroughly investigated [20,21].

The most commonly used drills for the machining of dental implants made of titanium and its alloys are fine-grained tungsten carbide drills, which are applied for a time ranging from 10 to 30 s, or embedded diamond drills, which are applied for the same amount of time. This machining operation is carried out under water irrigation at 20 °C. After the most aggressive machining, polishing is performed with coarse-grained silicon carbide polishing drills and then with fine-grained silicon carbide polishing drills [22,23,24]. Currently, zirconia drills are starting to be used instead of tungsten carbide or diamond inlay drills, but they do not have significant application due to their ease of fracture and high cost [25]. Therefore, it is important to determine the advantages and disadvantages of tungsten carbide and diamond drills.

In this research work, we tried to determine the influence of the type of drill on the production of mechanization, and we used the two most common ones. Tungsten carbide drills and diamond drills, as well as the direction of rotation of the drills on the dental implant, have been studied. The surface wettability, hardness, open-circuit corrosion potential, and potentiodynamic corrosion resistance have been characterized, along with the kinetics of metal ion release. Cytotoxicity studies of dental implant surfaces have been performed with human fibroblasts. This work is intended to help improve implantoplasty protocols so that they cause as little damage as possible to the tissues and to the long-term behavior of dental implants, and to this end, the following null hypotheses were proposed: (i) the type of bur used (diamond or tungsten carbide) does not influence the surface properties, corrosion resistance, or cytotoxicity of the machined Ti6Al4V surfaces, and (ii) the direction of rotation (clockwise or counterclockwise) has no effect on the surface modifications or biological outcomes.

## 2. Materials and Methods

**Figure 1:**
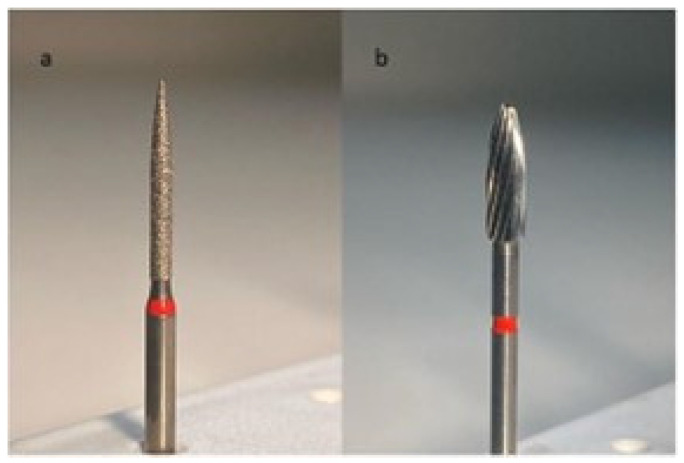
Milling tools used for the implantoplasty. (**a**) Diamond drill, (**b**) WC-drill.

A total of 190 disk-shaped samples with parallel faces, 10 mm in diameter and 3 mm in thickness (Klockner, Escaldes Engordany, Andorra), were studied. Control samples in the as-received condition were characterized, along with four additional sample sets corresponding to distinct implantoplasty procedures. The implantoplasty procedure was carried out by a single dental clinician (E.P.G) to ensure consistency in the treatment protocol and to reduce variability in both surface finish and experimental outcomes. The procedure was carried out using a force-controlled dynamometer to apply consistent pressure between the drill and the titanium surface. The applied force was maintained at a constant load of 10 N using an automatic dynamometric control system, corresponding to the mean force measured by 59 clinicians at the Universitat Internacional de Catalunya’s University Dental Clinic. The application of the load lasted 60 s for each disk. The surface of the samples was sequentially modified by using a GENTLEsilence LUX 8000B turbine (KaVo Dental GmbH, Biberak, Germany) with continuous irrigation, using either a coarse-grained diamond bur (ref: 863-010F-FG, NTI, Kahla, Germany) or a fine-grained WC-bur (ref: H48-L-014-FGXXL, NTI, Kahla, Germany) as milling tools. The drills can be observed in Figure 1. 

The influence of the direction of rotation of both diamond and tungsten carbide drills on the properties of Ti6Al4V surfaces was studied. This aspect is often taken into account by clinicians in their treatments considering that counterclockwise directions present lower abrasively and therefore reduce surface damage [26]. This fact is studied in this research to provide a scientific basis for this tradition among some professionals.

The samples studied included five groups:

- Control: Ti6Al4V disks machined.
- DV: Diamond drill with clockwise rotation.
- DCV: Diamond drill with counterclockwise rotation.
- TV: Tungsten carbide with clockwise rotation.
- TCV: Tungsten carbide with counterclockwise rotation.

The titanium surfaces were washed with distilled water twice and with acetone for 15 s and then air dried. The total number of samples was as follows: 5 groups × (3 samples (roughness) + 5 samples (wettability and surface energy) + 5 samples (hardness) + 5 samples (residual stress) + 10 samples (corrosion) + 5 samples (ion release) + 5 samples (cytotoxicity)) = 190 samples.

### 2.1. Morphology Charaterization

The design and surface quality of the drills were observed by scanning electron microscopy (SEM) using a Phenom XL Desktop microscope (PhenomWorld, Eindhoven, The Netherlands). The potential used was 20 keV, and different working distances were used. For the determination of the nature of the particles and surface phases, X-ray energy-dispersive microanalysis (Oxford 3200, Oxford, UK) was used.

### 2.2. Roughness

Surface roughness characterization was performed using CLS microscopy with an OLS Olympus Lext 3000 system (Olympus, Shinjuku, Japan). Prior to measurement, instrument calibration was verified using a precision reference specimen (SR 15) (Mitutoyo, Elgoibar, Spain). For each surface type, three independent measurements were performed on three distinct specimens. 

### 2.3. Wettability

Surface wettability was evaluated on five distinct specimens for each group through static contact angle measurements using the sessile drop technique. To determine the surface free energy components, both a polar liquid (ultrapure distilled water) and an apolar liquid (formamide) were used, following the Owens–Wendt approach. All measurements were performed under standardized environmental conditions, maintaining an ambient temperature of 22 ± 1 °C and relative humidity of 50 ± 5%, in order to minimize variability and ensure the consistency of the study. Contact angle measurements were carried out employing a contact angle goniometer (OCA15plus, Dataphysics, Filderstadt, Germany). Data analysis was performed using the SCA20 software package version 3.1 (Dataphysics, Filderstadt, Germany). Using a micrometric syringe, droplets were dispensed onto the sample disks using the sessile drop method. All measurements were conducted using a consistent microdroplet volume of 3 μL and a controlled flow rate of 200 μL/min, regardless of the liquid type (distilled water or formamide).

### 2.4. Nanoindentation

The mechanical characterization of the samples involved hardness determination using nanoindentation techniques. Hardness was assessed for the 5 groups, with five samples for each surface and 5 measurements for each sample. The hardness of the implant was measured with an iMiro system (KLA Tencor, Milpitas, CA, USA), while the hardness of the metal debris was determined using an MTS Nanoindenter model XP (MTS, Oak Ridge, TN, USA). Nanoindentation tests were performed using a Berkovich-type pyramidal indenter geometry under a constant strain rate of 0.05 s^−1^.

### 2.5. Residual Stress

For the determination of compressive residual stresses, five samples from each of the groups were used. The shallow X-ray methodology was used to determine the differences between the interatomic distances of the most superficial atoms. By means of the Bragg equation, we can determine these distances and compare them with the interatomic distances without surface tension. Having determined these differences we can calculate the microstrain, and with the value of the elastic modulus, we can calculate the residual stresses, which have an influence on many physicochemical properties of the surface [27,28].

Residual stress determination was performed using X-ray diffraction techniques, employing a diffractometer configured in Bragg–Brentano geometry (D500 model, Siemens, Munich, Germany). Measurements were conducted on the {213} crystallographic planes, which diffract at 2θ = 139.5°. The (EC) elastic constant of titanium along this crystallographic direction (90.3 ± 1.4 GPa) was calculated with Equation (1) for these crystallographic planes. Eleven ψ tilt angles were analyzed: 0°, along with five positive and five negative values. The peak positions were fitted using a pseudo-Voigt function with WinplotR software version 2.0 (freely available), and subsequently converted to interplanar spacings (dψ) through Bragg’s law [25]. Graphs of dψ versus sin^2^ψ were generated, and the slope (A) was determined through linear regression analysis using Origin software version 4.1 (OriginLab, Northampton, MA, USA). Residual stress (σ) was calculated according to Equation (2), where d_0_ represents the interplanar spacing at ψ = 0°.

(1) $$\mathrm{EC}=\frac{E}{1+\nu }$$

(2) $$\sigma =\mathrm{EC}\;\frac{A}{d0}$$

### 2.6. Corrosion Resistance

A total of 50 samples were tested for corrosion, with *n* = 10 samples allocated to each group. The constant exposed test area of each sample measured 19.6 mm^2^. Hank’s solution (ThermoFisher, Madrid, Spain) (Table 1) was used as the electrolyte in all experiments, as it closely mimics the ionic composition of human serum [26,27,28,29].

The electrochemical cell employed for corrosion testing consisted of a sterile 200 mL PP (polypropilene) recipient sealed with a PMMA lid, featuring six ports to accommodate the reference electrode, the counter-electrode, and the sample as the working electrode. A saturated calomel electrode (SCE, saturated KCl), with a potential of 0.241 V relative to the standard hydrogen electrode, was employed as the reference electrode in both open-circuit potential measurements and potentiodynamic tests. A platinum electrode was used as the counter-electrode in both open-circuit potential measurements and potentiodynamic tests [28,29,30,31,32]. All experiments were carried out at room temperature within a Faraday cage to prevent interference from external electric fields.

Open-circuit potential (OCP) measurements were performed using a two-electrode configuration, with the sample serving as the working electrode as well as the reference electrode. All tests were conducted over a 5-h period, with potential readings recorded every 10 s. In accordance with ASTM G31 standard specifications [33], the potential was considered stabilized when its variation remained below 2 mV over a 30-min interval. This test allows the assessment of the relative nobility of materials, with higher potentials indicating greater corrosion resistance. Data acquisition and E–t curve plotting were carried out using PowerSuite software version 2.1.

Cyclic potentiodynamic polarization tests were conducted using a three-electrode setup, where the sample functioned as the working electrode, while the reference and counter-electrodes were appropriately positioned within the electrochemical cell. Polarization curves were recorded for all sample groups in accordance with ASTM G5 standard specifications [34,35,36]. During the experiment, the potentiostat imposed a varying potential between the working (sample) and reference electrodes, thereby inducing a current flow between the working and counter-electrodes. These measurements were carried out immediately and consecutively after the completion of the 5-h open-circuit potential (OCP) stabilization period. Following potential stabilization, the potentiodynamic scan was initiated, applying a cyclic sweep from −0.8 V to +1.7 V relative to the stabilized open-circuit potential (EOCP), at a constant current flow of 2 mV/s. All test parameters were programmed into the PowerSuite software (version 2.1) using the PowerCorr-Cyclic Polarization module, which provided both the polarization curves and key corrosion parameters, including Icorr (corrosion current, μA), jcorr (corrosion current density, μA/cm^2^), Ecorr (corrosion potential, mV), and Rp (polarization resistance, Ω).

The corrosion potential (Ecorr) and corrosion current density (icorr) were determined by extrapolating the Tafel slopes. These slopes were further utilized to calculate the Tafel constants βa and βc, corresponding to the anodic and cathodic branches, respectively. Following the ASTM G102 standard guidelines [35,36,37,38,39], these coefficients were utilized to quantify the polarization resistance (Rp) through the Stern–Geary equation, which was subsequently employed to estimate the corrosion rate (CR), expressed in mm/year [36,37,38,39,40].


(3) $$Rp=\frac{\beta a\beta a}{2.303\;(\beta a+\beta c)\;icorr}$$

(4) $$\mathrm{CR}=K_{1}\;\frac{icorr}{\rho }\mathrm{EW}$$

K, the Stern–Geary constant calculated from the Tafel slopes, is rarely measured directly and is commonly assumed to have a standard value of 0.025. The equivalent weight (EW) is defined as the atomic weight (AW) divided by the number of electrons involved in the electrochemical reaction, a calculation typically applied to elemental metals. Rp refers to the polarization resistance, which reflects the material’s resistance to corrosion under small perturbations in potential.

### 2.7. Assessment of Ion Release from Samples

The evaluation of metal ion release from Ti6Al4V specimens into the physiological medium was conducted in accordance with ISO 10993-12 guidelines [41], using Hank’s solution as the release medium (Sigma-Aldrich, Co Life Science, St. Louis, MO, USA) (Table 1).

In accordance with the standard, a weight-based adjustment ratio was applied, utilizing 1 mL of medium per 0.2 g of sample weight. The final volume of medium was adjusted to 5 mL for each individual test specimen.

Five extraction time points were established—1, 3, 7, 14, and 21 days—during which the samples were incubated at a constant temperature of 37 °C to simulate prolonged exposure conditions and assess time-dependent ion release. The number of specimens used was 5 for each group. Then, 2.5 mL of solution was extracted at each analysis time point and replaced with an equal volume of fresh solution. The dilution effect of the release medium was accounted for and corrected to accurately determine the actual initial and final concentrations at each sampling point. The 2.5 mL extracted at each time point was divided into five aliquots for ICP-MS analysis, allowing for the calculation of representative mean values and standard deviations. The liquid in contact with the samples was carefully collected and then filtered using a 0.22 µm pore size membrane filter to remove any particulate matter. All medium extracts obtained during the study were acidified with 2% ultrapure nitric acid (HNO_3_ 69.99%, Suprapur, Merck, Darmstadt, Germany), to prevent the precipitation of metal ions in solution, before proceeding with the analysis. Samples were analyzed by inductively coupled plasma mass spectrometry (ICP-MS) using a Perkin Elmer Elan 6000 instrument (Perkin Elmer Inc., Waltham, MA, USA), enabling quantitative multi-elemental analysis with a detection limit of 1 ppt for approximately 90% of the elements.

### 2.8. Procedure for Cytotoxicity Testing

The cytotoxicity of the sample was evaluated by indirect exposure determination according to ISO 10993 [41]. Cytotoxicity assays were performed in triplicate (n = 5) for each of the five experimental groups, using cells cultured directly on the plate as the positive control and cell-free medium as the negative control.

Samples were handled aseptically throughout the assay. The cytotoxicity test consists of evaluating the percentage cell survival of a known cell line when exposed to a medium that has been in contact with a given material during 72 h of exposure at an incubation temperature of 37 °C. In this case, an indirect contact cytotoxicity test was performed according to the guidelines specified in ISO 10993-5, “Biological evaluation of medical devices”, part 5, “In vitro cytotoxicity tests”. To quantify cytotoxicity, the cell survival rate, which indicates cytotoxicity if <70%, was calculated.

The implantoplasty process generates the release of residues, not only from the metal of the implant but also from the drills used. In the latter case, the values are lower due to the hardness and tenacity of the drills with respect to titanium and its alloys. These particles will come into contact with the gingiva and soft tissues, and it is for this reason that we used human fibroblasts to observe their cytotoxic behavior. The HFF-1 line (ATCC^®^ SCRC-1041, LGC, Wesel, Germany) was used. These cells were maintained at −180 °C using dimethyl sulfoxide to keep them cold. Periodically, determinations were made to verify the absence of mycoplasma. Cultures were performed through incubation in the presence of 5% CO_2_. For HFF-1 culture, McCoy’s Medium (Thermo Fisher Scientific, Waltham, MA, USA) and Dulbecco’s Modified Eagle Medium (DMEM; Thermo Fisher Scientific, Waltham, MA, USA) supplemented with 10% of their own medium were used by adding 10% fetal bovine serum (FBS; Thermo Fisher Scientific, Waltham, USA), 1% L-glutamine (Thermo Fisher Scientific, Waltham, MA, USA), and 1% penicillin/streptomycin (Thermo Fisher Scientific, Waltham, MA, USA). The culture media were kept refrigerated (4 °C).

The samples to be analyzed (“extracts”) were prepared and tested according to the procedure described in the ISO 10993-5 standard. At a constant temperature of 37 °C, the material was incubated for 72 h in supplemented medium at a ratio of 1 mL per 0.2 g of sample. Cells were seeded at a density of 2 × 10^4^ cells/mL and incubated for 24 h prior to exposure to the sample extracts. Subsequently, cells were treated for 24 h with undiluted extract as well as 1:2 and 1:10 dilutions, prepared using complete medium. Cells were inspected for adhesion and morphology before and after contact with the extracts. Once the assay was completed, cells were lysed with Mammalian Protein Extraction Reagent (mPER). Cell viability was evaluated based on lactate dehydrogenase (LDH) enzymatic activity using a commercial assay kit (Roche Applied Science, Penzberg, Germany). The assay was performed according to the manufacturer’s instructions, and absorbance was measured at 492 nm to quantify LDH release as an indicator of cell membrane integrity and viability.

### 2.9. Statistical Analysis

The results were entered into a database (Microsoft Excel^®^, Redmond, WA, USA) and statistically processed using Stata 14 software (version 2.0) (StataCorp^®^, College Station, TX, USA). The data were processed with Student’s t-analysis, one-way ANOVA, and Tukey’s multiple comparisons tests to obtain possible statistical significance. A *p* < 0.05 was assigned as the criterion for statistically significant differences.

## 3. Results

**Figure 2:**
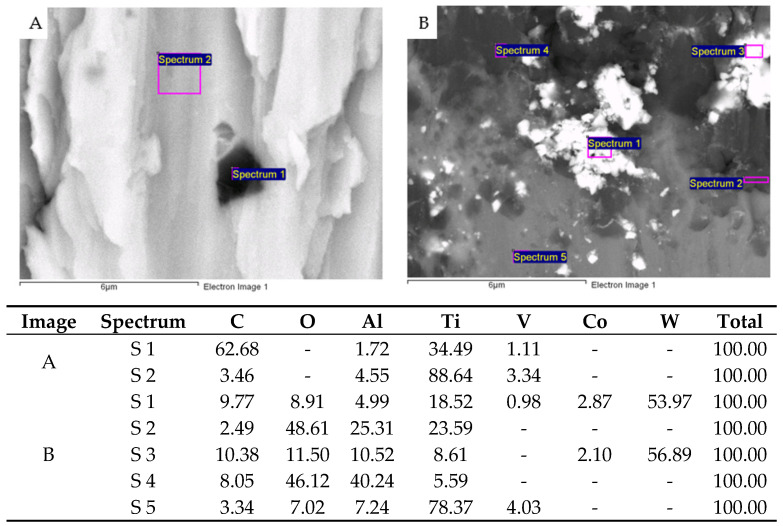
SEM micrographs of milled titanium disks highlighting surface residues. Semi-quantitative EDS compositional analysis at selected points: (**A**) diamond debris, (**B**) WC debris. The hyphen (-) indicates the absence of this chemical element in the surface residue.

**Figure 3:**
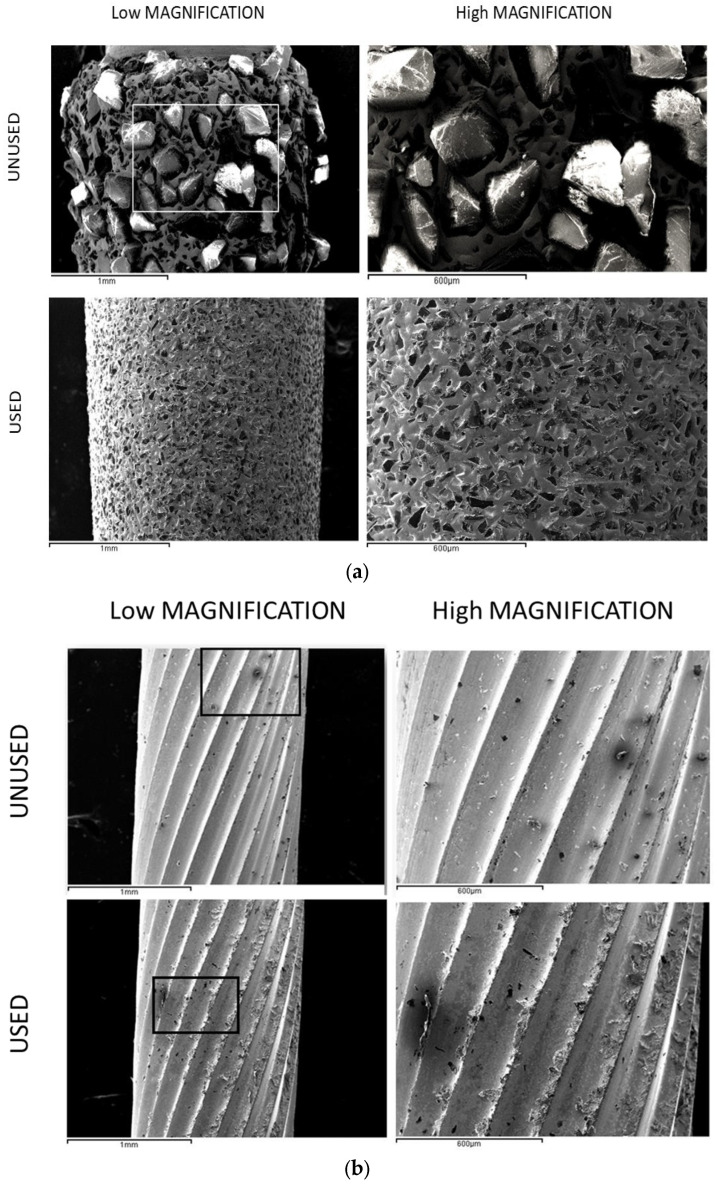
SEM micrographs at different magnifications of the drills used in this study, shown in their as-received condition and after use: (**a**) Diamond drilll and (**b**) tungsten carbide.

Figure 2 shows the surfaces of the Ti6Al4V disks after machining with the different milling drills and in the different directions studied. In Figure 3 we can observe the original unused drills under the electron microscope, including a diamond drill (Figure 3a) and a tungsten carbide drill (Figure 3b), respectively. In Figure 3 the same drills can be observed once they have been used for 15s, showing the absence of diamond particles detached from the drills and the tungsten carbide wear of the cutting edges of the drills.

X-ray microanalysis was performed on some impurities on the surfaces of the Ti6Al4V alloy machined with diamond drills (Figure 2) and it can be observed that in the areas of white inclusions, there are very high values of carbon coming from the diamond of the burr. Figure 2B shows images obtained from the microanalysis performed on an inclusion of the surface machined by the tungsten carbide drills, and it can also be observed that there is tungsten present on the surface that remains as a residue of machining.

**Figure 4:**
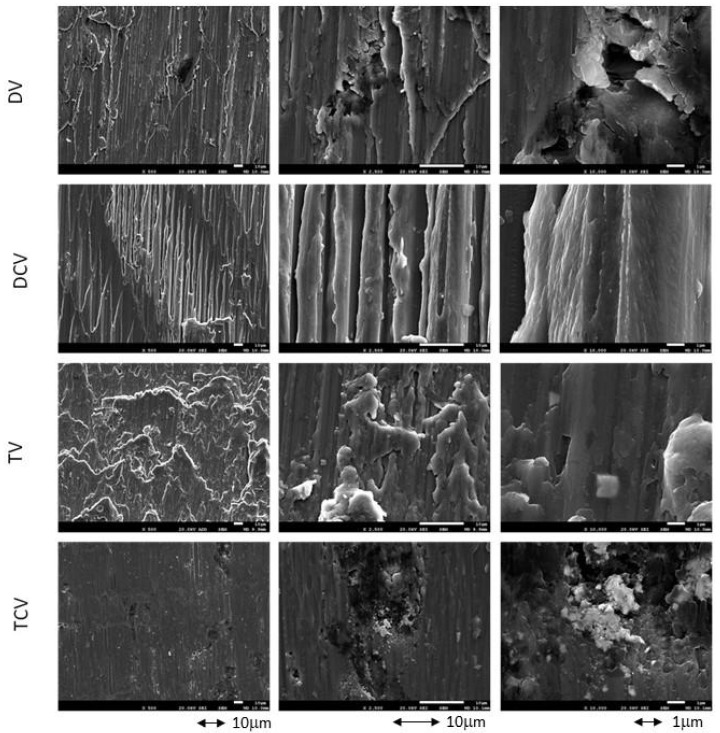
SEM micrographs at different magnifications showing the surface of titanium samples processed with different milling protocols using both types of drills.

Table 2 shows the roughness values with the different milling cutters, showing that the roughness increases in a statistically significant way, with *p* < 0.005, between the control and the diamond and tungsten carbide milling cutters, as well as between the two types of milling cutters. Figure 4 shows the different Ti6Al4V surfaces treated with different drills. It can be seen that machining causes a decrease in the contact angle and makes the surfaces more hydrophilic. The hardness values of the surfaces treated with implantoplasty with respect to the control surfaces produce a higher hardness value due to plastic deformation with material removal, which is also confirmed by the higher values of compressive residual stress of the machined surfaces. These results show that the deformation severity is higher for the samples machined with the diamond drills as they cause a higher roughness, and the compressive residual stresses are statistically significantly higher, with *p* < 0.005, than those machined with tungsten carbide and, of course, with the control. This higher compressive residual stress causes higher surface hardness of the diamond-machined ones.

It can be observed that the values of the properties studied do not have a statistically significant influence on whether the direction is clockwise or counterclockwise. In the case of the diamond drill, since it is a cylinder without a pattern, it is clear that it will not have any influence, but an influence is not observed in the case of the tungsten carbide drill either, for which, although it has a fillet design, the deformation and mechanical properties obtained are very similar.

**Figure 5:**
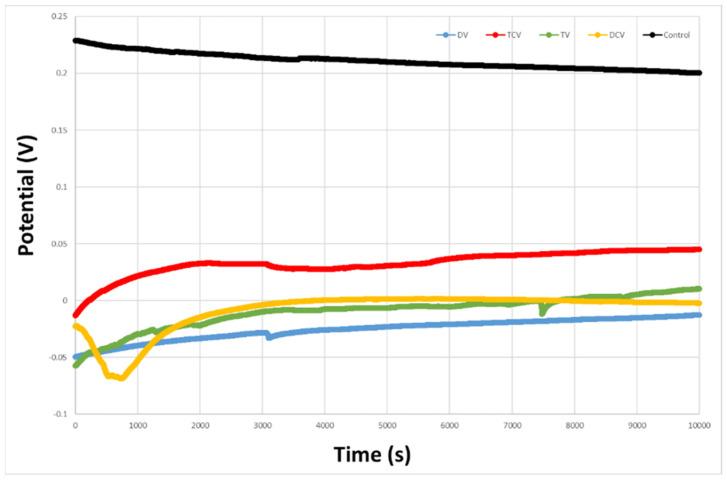
Representative open-circuit potential (OCP) vs. time curves for the different machined surfaces (DV, DCV, TV, TCV).

The natural or open-circuit potential (EOCP) is defined as a lack of current flow between the electrode and the metal, i.e., the reaction kinetics between the cathodic and anodic zones are equalized. This potential estimates the corrosion of the studied sample, which is a material that favors corrosion (active) or is inert to corrosion (passive) [42,43]. The obtained values of natural corrosion potential (EOCP) are illustrated in Figure 5. The Ti6Al4V control samples exhibited a higher average corrosion potential compared to the implantoplasty-treated samples, as shown in Table 3. This more electropositive behavior indicates greater thermodynamic stability and, consequently, higher corrosion resistance. 

**Figure 6:**
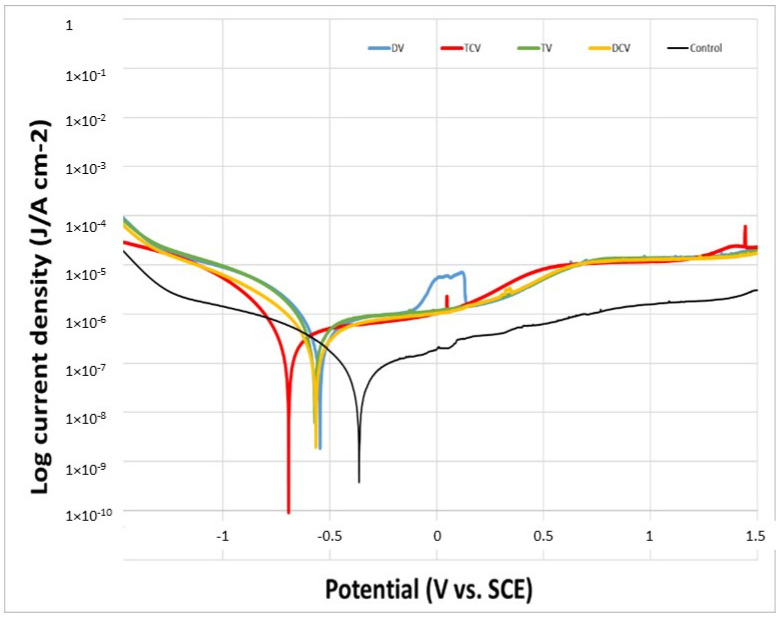
Potentiodynamic polarization curves of the various machined surfaces (DV, DCV, TV, TCV).

The potentiodynamic polarization curves are shown in Figure 6. Across all evaluated electrochemical parameters, the control group demonstrated superior corrosion resistance compared to the implantoplasty-treated groups, whether machined with diamond or tungsten carbide burs. Specifically, the control samples exhibited lower values of corrosion potential (E_CORR_) corrosion current density (I_CORR_), and corrosion rate (CR), along with higher polarization resistance (Rp), indicating enhanced stability in the corrosive environment (Table 3).

The corrosion resistance parameters, evaluated through both open-circuit potential and potentiodynamic polarization studies, showed no statistically significant differences between the rotational directions for any of the drilling techniques assessed; this was expected since, as we have seen in the results of Table 3, there are no changes in the physicochemical properties studied on the different surfaces. 

**Figure 7:**
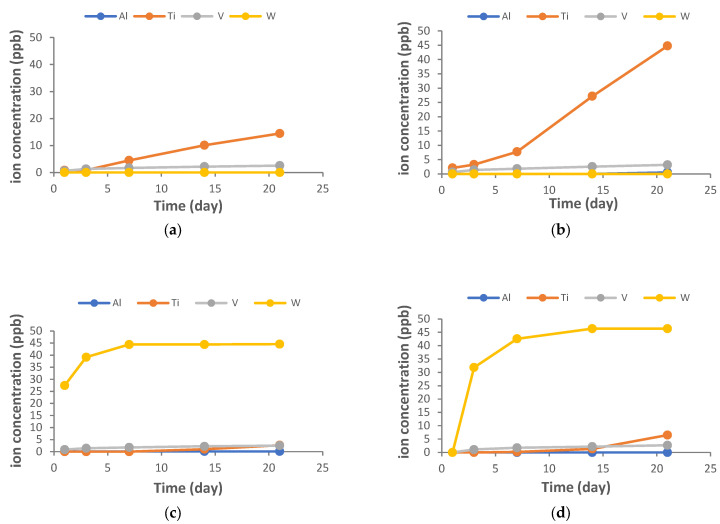
Time-dependent release profiles of Ti, Al, V, and W ions from various machined surface types: (**a**) DV, (**b**) DCV, (**c**) TV, (**d**) TCV.

Figure 7 shows the ion release detected at the different study times. There are no differences in the direction of rotation of the drills, but there is a very significant release of tungsten in the CW drills.

**Figure 8:**
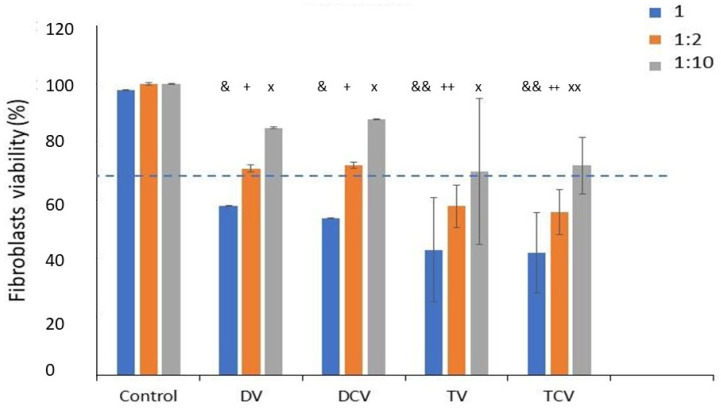
Fibroblast cell viability (percentage) for the different machined surfaces. Values for each dilution accompanied by a single symbol denote statistically significant differences compared to values without a symbol (*p* < 0.05). A double symbol indicates statistically significant differences relative to values without a symbol or with a single symbol, also at *p* < 0.05. The dashed line indicates 70% fibroblast survival which indicates the limit of cytocompatibility.

The results of the cytotoxicity tests, presented as the percentage of live cells relative to the control group (disks without implantoplasty), are presented in Figure 8. All tested sample groups subjected to implantoplasty procedures exhibited cytotoxic effects in human fibroblast assays when exposed to the undiluted extract (1:1), indicating a reduction in cell viability under these conditions.

It can be observed that the values indicating the release of ions are low, in the order of parts per billion. However, the results of the presence of tungsten generated by the tungsten carbide drills in the implantoplasties are noteworthy. The values are significant, with linear growth during the first days and then a stabilization of the release.

The results shown in Figure 8 correspond to the cytocompatibility of the materials with fibroblasts in different solutions during the implantoplasty processes. These show that the solutions generated in the implantoplasty, on the surfaces machined both with a diamond drill and with tungsten carbides, have a cell viability of less than 70% and can therefore be considered cytotoxic solutions. When the solutions are diluted by half, the solutions of the surfaces machined with diamond drills have a viability slightly above 70% and can be considered cytotoxic, which is not the case for the solutions from the machining with tungsten carbide drills, where the cell viability is close to 60% but does not reach the limit of cytotoxicity, which is 70% of live cells according to the standard. At 1:10 dilutions, all solutions show cell viability and can be considered cytocompatible. These results highlight the need for abundant irrigation with water on titanium surfaces on which implantoplasty has been performed, especially those machined with tungsten carbide.

## 4. Discussion

Implantoplasty procedures are becoming more and more common due to the fact that 24% of dental implants suffer from peri-implant diseases. Many clinicians perform this procedure due to the ease with which it can be performed, avoiding the replacement of the dental implant, which in many cases involves a long process of tissue regeneration and the repositioning of the dental implant [9,10]. The use of this technique does not have a protocol defined or agreed upon by the health authorities or scientific societies. However, very abrasive burs such as diamond or tungsten carbide burs are generally used to remove the dental implant files and thus the bacterial biofilm. This coarser machining is refined by the subsequent use of smaller-grained silicon carbide burs, and Arkansas type burs can finally be used in order to reduce the surface roughness of the titanium or its alloys. Therefore, this contribution aims to determine the advantages of one or the other milling cutter in the initial machining, since the rest of the process seems to be under general procedural agreement.

From the results obtained it can be seen that diamond drills are more abrasive than tungsten carbide drills. It can be seen that after only one minute of machining of the Ti6Al4V surface, abrasive diamond particles are released into the physiological environment. Therefore, the lifetime of this type of drill is short, which should be taken into account by the clinician [44,45,46,47]. The higher abrasive capacity of diamond generates a higher roughness (from 0.22 (control) to 0.73 μm) and also a higher nano-hardness (from 2.2 GPa to 4.8 GPa), which is corroborated by X-ray diffraction studies with higher values of compressive residual stress from −26 MPa to −125 MPa. An interesting result from a biological point of view is that machining generates a higher hydrophilic capacity, as already described by Pereira et al. [48]. The residual stresses generate a lower contact angle, and this increases the interface between the blood and the titanium surface, which will increase the adsorption of proteins on the surface and, in principle, should lead to higher biological activity [49,50,51,52]. 

The corrosion results clearly show a considerable loss of resistance with respect to the control surface both in the open-circuit and potentiodynamic tests. The presence of compressive stresses on the material surface induces the formation of localized anodic and cathodic sites, which act as electrochemical cells. This heterogeneity in stress distribution facilitates preferential corrosion at the interfaces between regions experiencing tensile and compressive residual stresses, thereby accelerating localized degradation processes [53]. The results of the surfaces milled with diamond and tungsten carbide drills show that those machined with the latter drills have worse performance against electrochemical corrosion. This fact might seem contradictory, since diamond drills are more abrasive and have a higher residual stress, and therefore, the corrosion resistance should be smaller. The justification that corrosion is favored for samples machined with tungsten carbide is that the carbides remaining on the surface of Ti6Al4V cause oxidation of the carbides, and this justifies these values [54,55]. The same behavior was observed when the implants were shot-blasted to obtain rough surfaces with abrasive particles of silicon carbide compared to those shot-blasted with silicon oxides or aluminum oxides. In the first case, the dental implants had higher corrosion resistance values than the oxides due to the process of carbide oxidation. However, it can also be observed that the results pertaining to mechanical properties do not show a significant difference in corrosion resistance. 

Ion release analyses have revealed the continuous release of tungsten ions into the physiological environment. Despite their low concentration at the parts-per-billion level, the sustained upward trend observed over time necessitates thorough evaluation due to its potential biological implications [56]. The toxicological effects of the ingestion of tungsten ions are not clearly known, although there are different projects under development on the possible carcinogenic effects of these ions [57,58]. 

Cytocompatibility assays performed using indirect cytotoxicity tests revealed slightly below 70% viability for the 1:1 and 1:2 dilutions, which are indicative of cytotoxic effects. In contrast, the 1:10 dilution showed a marked reduction in cytotoxicity toward fibroblasts. Although the mean cell viability for the 1:10 dilution was slightly above the 70% threshold—technically classifying it as non-cytotoxic—the result remains questionable due to the high standard deviations observed. This variability suggests that some individual cultures exhibited cytotoxic responses, thereby casting doubt on the overall biocompatibility of the extract [41]. It has been determined that the particles that become detached in the processes of implantoplasty, caused by mechanization of the implant, cause a decrease in their metabolic activity in human fibroblasts after the tenth day. The studies were performed on Ti6Al4V [59].

Particles released during implantoplasty show increased hardness and corrosion rates due to fracture stresses and high internal energy, with cytotoxic oxides causing black discoloration [60,61]. Their retention in tissues, especially nanoparticles with unknown biological responses, poses a concern, while larger particles are usually encapsulated and cleared by macrophages [62,63,64,65,66].

In implantoplasty, particle dispersion should be minimized using protective barriers to enable effective removal. Proper drill selection and abundant irrigation are essential to avoid cytotoxic tungsten ion accumulation. Given these risks, implantoplasty requires caution, and maintaining oral hygiene remains key to preventing peri-implantitis.

In summary, Table 4 presents the key findings, showing that diamond drills, when operated under the same mechanical load as tungsten carbide drills, produce greater abrasiveness. This results in increased surface roughness, elevated hardness, higher compressive residual stresses, and an accelerated corrosion rate. These effects are attributed to the increased surface instability caused by the higher surface energy generated during machining. On the other hand, the higher surface energy of the surfaces treated with diamond drills leads to lower contact angles and, consequently, better wettability, which will be important for improving the subsequent biological behavior. Tungsten carbide drills show worse ion release behavior due to the presence of tungsten and therefore also lower cytocompatibility.

It is common for clinicians to use tungsten carbide burs at the beginning of treatment due to their higher abrasiveness and therefore ease of machining, but based on our in vitro results, where diamond burs provide a more hydrophilic final surface with a lower degree of cytotoxicity, it is advised that these drills be applied at the end of the treatment. A final irrigation with physiological saline solution is always very important. However, the clinical efficacy of this approach should be confirmed by additional studies.

## 5. Conclusions

Implantoplasty on Ti6Al4V dental implants using diamond drills resulted in higher surface roughness (increasing from the control value of 0.22 to 0.73 μm), hardness (from the control value of 2.2 to 4.8 GPa), compressive residual stress (from the control value of −26 to −125 MPa), and wettability compared to tungsten carbide drills. The contact angle for the control was around 80°, and was 62° and 54° for tungsten carbide and diamond drills, respectively. However, the surfaces treated with a diamond drill showed higher corrosion rates of 0,0518 mm/year in relation to 0.0323 and 0.0044 mm/year for the surfaces treated with tungsten carbide and the control, respectively. No statistically significant differences were detected with respect to the rotational direction of the drills. Cytotoxic effects on human fibroblasts were detected for both drill types, with a higher incidence in tungsten carbide drills and at lower dilutions. From the ion release results, we can observe the presence of tungsten in the solution. These findings underscore the importance of careful clinical application of the implantoplasty technique.

## Figures and Tables

**Table 1:** Chemical composition of Hank’s solution expressed in mM.

Component	K_2_HPO_4_	KCl	CaCl_2_	Na_2_HPO_4_	NaCl	NaHCO_3_	MgSO_4_	C_6_H_12_O_6_
Composition	0.44	5.4	1.3	0.25	137	4.2	1.0	5.5

**Table 2:** Roughness, contact angle, hardness, and residual stress of samples tested.

Scheme	Ra (μm)	Contact Angle (°)	Hardness (GPa)	Residual Stress (MPa)
Control	0.22 ± 0.09	80 ± 7	2.2 ±1.2	−26 ± 5
DV	0.73 ± 0.10 *	54 ± 5 *	4.8 ±1.0 *	−125 ± 12 *
DCV	0.72 ± 0.08 *	56 ± 5 *	4.7 ±0.9 *	−124 ± 20 *
TV	0.59 ± 0.09 **	62 ± 9 **	3.9 ±0.9 *	−111 ± 15 *
TCV	0.57 ± 0.12 **	67 ± 8 **	3.9 ± 1.2 *	−112 ± 10 *

Values marked with an asterisk (*) indicate statistically significant differences (*p* < 0.05) compared to those without an asterisk. When the asterisk is double (**) it means that the results have statistically significant differences with respect to those without an asterisk (*) or those with only one asterisk, with a *p* < 0.05.

**Table 3:** Roughness, contact angle, hardness, and residual stress of tested samples.

Sample	EOCP (V)	jcorr (µA/cm^2^)	Icorr (µA)	Ecorr (mV)	Rp (µΩ)	CR (mm/year)
Control	−0.0204 ± 0.0010	0.067 ± 0.018	0.051 ± 0.007	−361 ± 14	1.142 × 10^12^ ± 1.135 × 10^11^	0.0044 ± 0.0009
DV	−0.0798 ± 0.0010 *	3.645 ± 0.654 *	2.843 ± 0.453 *	−511 ± 30 *	1.445 × 10^11^ ± 0.235 × 10^10^ *	0.0323 ± 0.0032 *
DCV	−0.0638 ± 0.0011 *	2.261 ± 0.123 **	1.764 ± 0.324 **	−512 ± 22 *	1.797 × 10^11^ ± 0.305 × 10^10^ *	0.0263 ± 0.0024 *
TV	−0.2356 ± 0.0052 **	4.435 ± 0.013 *	3.459 ± 0.202 *	−483 ± 83 *	1.412 × 10^11^ ± 0.245 × 10^10^ *	0.0518 ± 0.0046 **
TCV	−0.3749 ± 0.0064 **	1.791 ± 0.328 **	1.392 ± 0.433 **	−603 ± 134 *	1.193 × 10^11^ ± 0.396 × 10^10^ *	0.0515 ± 0.0085 **

Values marked with a single asterisk indicate statistically significant differences (*p* < 0.05) compared to those without an asterisk. Double asterisks indicate statistically significant differences (*p* < 0.05) compared to both unmarked values and those marked with a single asterisk.

**Table 4:** A summary of the results of the different parameters obtained by diamond or tungsten carbide in relation to the control.

Property	Diamond Drill	Tungsten Carbide Drill
Roughness	⇑⇑	⇑
Nanohardness	⇑⇑	⇑
Residual Stress	⇑⇑	⇑
Wettability	⇑⇑	⇑
Corrosion	⇑⇑	⇑
Ion Release	⇑	⇑⇑ (W)
Citotoxicity	⇑	⇑⇑
Where ⇑ = High; ⇑⇑ = Higher; W = Tungsten release		

## Data Availability

The original contributions presented in this study are included in the article; further inquiries can be directed to the corresponding author.
